# Enhancing the Piezoelectric Properties of 3D Printed PVDF Using Concurrent Torsional Shear Strain

**DOI:** 10.3390/polym15214204

**Published:** 2023-10-24

**Authors:** Pu Han, Alireza Tofangchi, Derek Carr, Sihan Zhang, Keng Hsu

**Affiliations:** 1 Ira A Fulton Schools of Engineering, Arizona State University, Tempe, AZ 85212, USA; pu.han@asu.edu; 2 J. B. Speed School of Engineering, University of Louisville, Louisville, KY 40208, USA

**Keywords:** PVDF, torsional shear strain, piezoelectric properties, 3D printing

## Abstract

Extrusion-based polymer 3D printing induces shear strains within the material, influencing its rheological and mechanical properties. In materials like polyvinylidene difluoride (PVDF), these strains stretch polymer chains, leading to increased crystallinity and improved piezoelectric properties. This study demonstrates a 400% enhancement in the piezoelectric property of extrusion-printed PVDF by introducing additional shear strains during the printing process. The continuous torsional shear strains, imposed via a rotating extrusion nozzle, results in additional crystalline β-phases, directly impacting the piezoelectric behavior of the printed parts. The effect of the nozzle’s rotational speed on the amount of β-phase formation is characterized using FTIR. This research introduces a new direction in the development of polymer and composite 3D printing, where in-process shear strains are used to control the alignment of polymer chains and/or in-fill phases and the overall properties of printed parts.

Material-extrusion-based 3D printing technology has become the most widely used additive manufacturing method for thermoplastic polymer materials due to its capability and cost-effectiveness [1,2]. It inherently introduces shear strain in the material, which stretches the polymer during deposition [3]. These stretches, however, can compromise the mechanical strength of the 3D printed parts [4]. As a result, various approaches, such as print parameter optimization [5,6,7], polymer material modification [8,9,10], in-process heating [11,12,13,14,15], and post-process treatments [16,17] have been explored to either mitigate stretches or modify materials to enhance the overall strength of printed objects. Remarkable progress has been made through these approaches. However, the impact of the stretching of polymer chains due to shear during deposition on the material properties is not always negative. Polymers such as polyvinylidene difluoride (PVDF), can actually benefit from stretches introduced to its polymer chains. In this semicrystalline polymer, one of the crystalline phases, β-phase, which is directly responsible for the piezoelectric property of PVDF, can see an increase in its concentration with the application of mechanical stretching [18,19]. Due to the piezoelectric property of this material, it can be used for multiple applications such as a pressure sensor [20], force sensor [21], nanogenerator [22], flow velocity sensor [23], humidity sensor [24], tactile sensor [25], and energy conversion [26]. Numerous works have been conducted to improve the β-phase concentration and the piezoelectric property, including mechanical stretching [27,28], the addition of nanofillers [29,30], poling [31,32], and heat treatment [33,34]. Curiously, the deliberate introduction and control of stretching in polymer materials using 3D printing techniques remains unexplored in the existing literature. In this study, we present our findings in the enhancement of the β-phase concentration and piezoelectric properties of PVDF through an innovative in-process stretching treatment employed in 3D printing.

To introduce and control polymer stretching during extrusion-based 3D printing, we implemented a distinctive spinning nozzle apparatus on a 3D printer (Makergear M2), as depicted in Figure 1a. The spinning nozzle can rotate at rates within a range of 50 to 1000 rpm. The relative movements between the nozzle introduces addition continuous torsional shear strains (or stretching of polymer chains) in the polymer melt in two locations: in the PVDF filament prior to melting in the feedstock passage through the nozzle and during deposition between the rotating nozzle and the stationary surface (build plate or an existing layer). To facilitate the nozzle rotation, a DC motor with a set of pulleys for speed control is used to drive the extruder assembly. The extruder assembly contains all the necessary components for the extrusion technique, including the nozzle, cylindrical heater block, and cooling fins for thermal control co-axially mounted and supported by two rotational bearings as shown in Figure 1a. Induction heating and a thermal couple in contact with the rotating nozzle surface is used for temperature control in the polymer melt during printing. During rotating-nozzle-assisted printing, the filament is fed through the extruder assembly and extruded from the rotary hot end onto a build plate, while the extruder assembly spins at a prescribed rate. For the control samples, all components remain stationary. In this case, the polymer melt inside the hot end exhibits a nozzle flow solely along the vertical direction, as illustrated in Figure 1b. However, with the rotation of the hot end, a torsional shear flow is introduced in the nozzle flow, leading to stretching of the polymer chains within the filament, as depicted in Figure 1c. The printed samples for β-phase concentration are displayed in Figure 1a on the build plate.

To investigate the effect of this tortional strain rate on the β-phase concentration in PVDF, we prepared seven sample groups with different nozzle spin rates ranging from 0 to 1062 rpm. All samples were printed with a 250 °C nozzle temperature and a 100 °C bed temperature at 10 mm/s, as recommended by the manufacturer (Nile Polymers). The β-phase concentration data were obtained using Fourier Transform Infrared Spectroscopy (FTIR). An increase in the β-phase concentration was observed as the nozzle spin rate increased (Figure 2). Specifically, at 1062 rpm, the concentration increased from 33% to 42% (Figure 2b). The authors attribute this increase to the continuous torsional shear strain generated by the friction between the material and the nozzle wall during 3D printing at two locations in the extrusion path: (1) within the heated chamber, where the part of feedstock transitioned from rigid solid state to polymer melt, and (2) at the nozzle exit, where the extruded material is sheared between the rotating nozzle and the stationary print surface. The underlying physics behind this increase is that stretching of the polymer melt leads to the alignment of the polymer chains, resulting in a higher β-phase concentration [18]. In the spinning nozzle process, the stretch on the polymer melt increases with the nozzle spin rate and, therefore, increases the chain alignment and the β-phase concentration.

In the control sample with no nozzle rotations, there is also inherent stretching of the material in two locations: where the cross-sectional geometries of the flow path transition from 1.75 mm ID to the nozzle orifice dimension at 0.4 mm ID and the 90-degree turn as the material exits the nozzle surface and lands on the print surface [12,15,35]. The stretching experienced by the material can also contribute to the alignment of the polymer chains along the direction of nozzle movement after deposition. On the other hand, in the printed sample with nozzle rotations, the shear rate inside the nozzle is approximately 40 times higher at 1000 rpm (for a 1 mm wide deposition at 10 mm/s) compared to that in the control sample because of the additional torsional relative movements of the inner walls and nozzle surfaces with respect to the stationary feedstock and print surface.

It is worth noting that an observably higher concentration of β-phase is observed on the top surface of the printed PVDF as compared with the bottom surface across all spin rates. The authors attribute this difference to the additional shear strain observed on the “inner” side of the 90-degree turn in the flow as it exits the nozzle, due to the much smaller free surface on the inner corner of the turn compared with the outer conner. This difference in local shear strain and, therefore, chain stretching is responsible for the difference in the β-phase concentration observed between the bottom surface (Figure 2a) and the top surface (Figure 2b) of the printed PVDF. 

Previous studies employing an electrospinning approach on PVDF did not result in any increase in piezoelectric properties due to the randomly distributed dipole orientations in the produced samples [18]. The main distinction in our approach is that the stretch in the material inside the nozzle occurs in a circular pattern, and as the material exits the nozzle and adheres to the build plate, the 90-degree turn aligns the stretch (polymer chains) vertically with respect to the nozzle’s movement direction (Y direction, assuming the X direction as the nozzle’s movement direction and Z as the build direction). This distinction offers the possibility of having aligned polar monomers in the polymer chains, which is crucial for the piezoelectric property.

To further evaluate the piezoelectric properties of the material printed using the spinning nozzle technique, six devices were prepared, as illustrated in Figure 3a. The devices depicted in Figure 3a consist of two layers of 3D printed PVDF with two layers of ultrasonically embedded copper wires as electrodes. The tooling setup for the test is illustrated in Figure 3b, while the testing apparatus, including the cyclic movement control and data acquisition, is shown in Figure 3c. All four spinning samples were printed at a spinning rate of 500 rpm. The voltage difference between the two layers of copper electrodes was monitored as the cyclic bending was introduced to the devices in a three-point bending configuration.

An increase in the voltage between the two copper electrode layers was observed in the device, where the PVDF was deposited with the spinning nozzle technique (Figure 4), indicating an overall better piezoelectric property in the material. Overall, 1.5- to 4-fold increases were observed between the control and the devices printed with the spinning nozzle. The authors suggest two main reasons for the significant improvement in piezoelectric voltage. The first reason is the increase in the β-phase concentration caused by the continuous torsional shear strain, as shown in Figure 2. However, the increase from 33% to 38% (at 500 rpm) in the β-phase concentration can hardly translate into the 400% improvement in piezoelectric voltage. The second reason is the increase in aligned dipoles due to this spinning technology. The dipoles in the β-phase show piezoelectric behavior but are usually randomly oriented in homogeneous PVDF material [19]. Therefore, most of the voltage bias generated in homogeneous PVDF due to material deformation is neutralized because of the randomized dipole orientation. As a result, the number of charges that can be collected by the copper electrode is reduced. This spinning technology increases the number of the aligned dipoles through the torsional shear strain and results in higher piezoelectric properties. The physics behind it is similar to the mechanical stretch method [19,27,28], but the work using in-process 3D printing technology has not been reported before. The difference in voltage between the four samples may be due to: (1) pre-existing surface defects from printing due to spinning that increase the surface roughness and result in less contact area; (2) weak bonding between the copper wire and PVDF in certain regions; (3) the position of the copper wires if at the boundary of two deposited tracks (results in less contact area). 

It is important to note that even though the conventional electric polling treatment can typically result in β-phase concentrations ranging from 42% to 56.8% [36], the spinning technique demonstrated here could reach a similar level of β-phase concentrations at even higher spin rates. Additionally, this approach opens up a new direction in extrusion-based 3D printing by utilizing induced shear strain for orientation purposes. This can be particularly useful for enhancing the crystallization of semicrystalline polymers or aligning carbon fiber infills or conductive fibers in different directions, although the direction of alignment is in a circular shape instead of along a certain direction. 

In conclusion, this work presents a novel approach utilizing an in-process spinning nozzle 3D printing technique to increase the β-phase concentration and piezoelectric property of PVDF. The results demonstrate an increase in the β-phase concentration from 33% to 42% at 1062 rpm, as well as a significant improvement of up to 400% in the piezoelectric property at 500 rpm in printed devices. By introducing shear strain during the printing process, this method holds the potential to not only boost polymer crystallization but also align infill materials, thereby improving properties such as piezoelectric, mechanical, and electrical. The significance of this research extends beyond these immediate outcomes. This research opens up new possibilities for advancing the performance of 3D printed materials and expanding their applications in various industries. This research highlights how manipulating shear strain during the printing process can bring about significant changes, pointing towards an exciting future for the development of 3D printing technology and its wide-ranging uses.

## Figures and Tables

**Figure 1:**
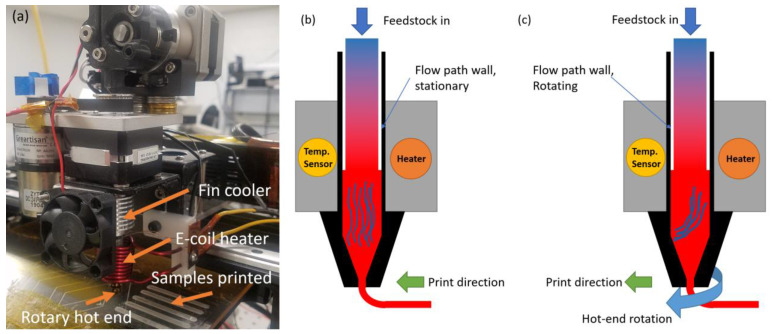
(**a**) Spinning nozzle experimental apparatus with contactless heater (*E. coil*) and printed sample on build plate. (**b**) Schematic diagram of nozzle region when it is stationary. (**c**) Schematic diagram of nozzle region when the nozzle is rotating. For (**b**,**c**), the blue curved lines inside the nozzle represent the predicted behavior of the polymer chains (plotted thousands of times larger to clearly show) due to the friction between the material and nozzle wall.

**Figure 2:**
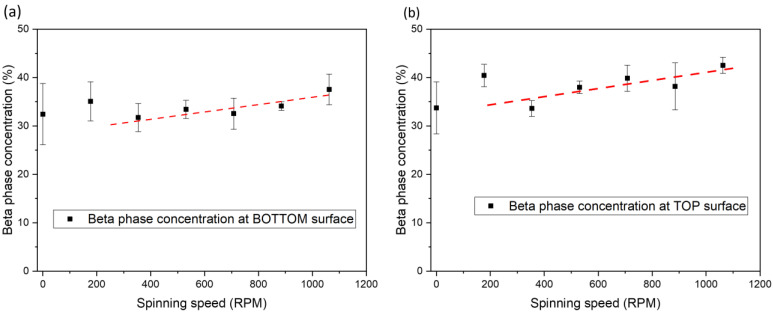
(**a**) Spinning nozzle experimental apparatus with contactless heater (*E. coil*). (**b**) Schematic diagram of nozzle (error bar is standard deviation).

**Figure 3:**
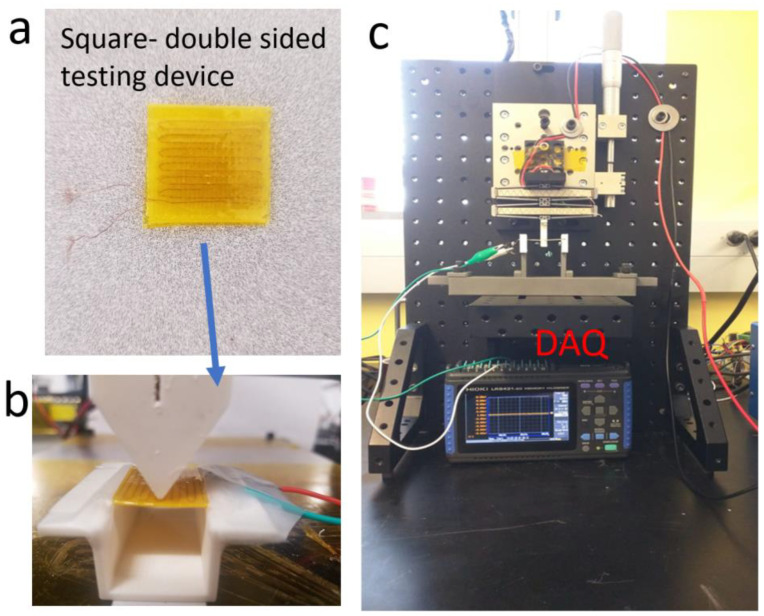
(**a**) Square-shape double-sided testing device with PVDF and ultrasound embedded copper wires. (**b**) Tooling setup. (**c**) Testing apparatus with vibration control and data acquisition device.

**Figure 4:**
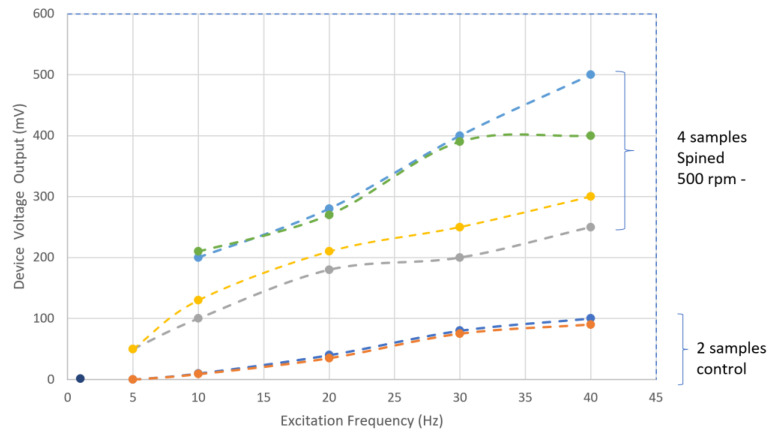
Piezoelectric property test: voltage output at a set of frequency for control and spinning samples.

## Data Availability

The data that support the findings of this study are available from the corresponding author upon reasonable request.
